# Involvement of galanin and galanin receptor 1 in nociceptive modulation in the central nucleus of amygdala in normal and neuropathic rats

**DOI:** 10.1038/s41598-017-13944-6

**Published:** 2017-11-10

**Authors:** Shi-Yang Li, Mei-Ling Huo, Xu-Yang Wu, Yu-Qing Huang, Lei Wang, Xin Zhang, Yan-Mei Jiang, Meng-Lin Zhang, Lin-Lin Wang, Long-Chuan Yu

**Affiliations:** 1 School of Pharmacy, Key Laboratory of Molecular Pharmacology and Drug Evaluation (Yantai University), Ministry of Education, Collaborative Innovation Center of Advanced Drug Delivery System and Biotech Drugs in Universities of Shandong, Yantai University, Yantai, 264005 P.R. China; 2 Neurobiology Laboratory, College of Life Sciences, Peking University, Beijing, 100871 P.R. China

## Abstract

The present study was performed to explore the role of galanin and galanin receptor 1 (GalR 1) in nociceptive modulation in the central nucleus of amygdala (CeA) in normal rats and rats with neuropathy, and the involvement of GalR 1 and PKC was also investigated. The hindpaw withdrawal latencies (HWLs) to thermal and mechanical stimulations were increased in a dose-dependent manner after intra-CeA injection of galanin in both normal rats and rats with neuropathy. The increased HWLs were significantly attenuated by intra-CeA injection of galanin receptor antagonist M40, indicating an involvement of galanin receptor in nociceptive modulation in CeA. Furthermore, intra-CeA administration of the GalR 1 agonist M 617 induced increases in HWLs in normal rats, suggesting that GalR 1 may be involved in galanin-induce antinociception in CeA. Additionally, intra-CeA injection of the PKC inhibitor inhibited galanin-induced antinociception, showing an involvement of PKC in galanin-induced antinociception in CeA of normal rats. Moreover, there was a significant increase in GalR1 content in CeA in rats with neuropathy than that in normal rats. These results illustrated that galanin induced antinociception in CeA in normal rats and rats with neuropathy, and there is an up-regulation of GalR1 expression in rats with neuropathy.

## Introduction

Galanin, consists of 29 or 30 amino acid in human, is a neuropeptide^1^. It is known that there are three kinds of galanin receptor subtypes, including galanin receptor 1 (GalR 1), galanin receptor 2 (GalR 2) and galanin receptor 3 (GalR 3), and all of these belong to G protein coupled receptors^2–5^. Galanin and its receptors are widely distributed in the peripheral nervous systems and central nervous systems^3,6,7^.

A lot of studies have reported that galanin and its receptors are involved in multiple physiological functions and pathological processing, such as learning and memory, modulating pituitary hormone release, energy homeostasis^3,4,6,8–12^. Previous studies have demonstrated that galanin and galanin receptors play important roles in the transmission and/or modulation of nociception at spinal levels^6,12^, as well as in the brain, such as hypothalamic arcuate nucleus, nucleus accumbens, periaqueductal grey, anterior cingulate cortex and the central nucleus of amygdale^11,13–24^.

The central nucleus of amygdala (CeA) is a very important brain structure, and there are high densities of galanin and galanin receptors found in the CeA^3,6,7,25^. Lots of studies demonstrated that CeA is involved in pain modulation^17,26–30^. Recent research work also demonstrated that CeA represents a convergence of pathway for pain, stress and emotion^30^. These studies are full proved that the CeA plays an important role in nociceptive modulation.

Previous study in our laboratory demonstrated that intra-CeA injection of galanin produced analgesia in normal rats, and both mu- and delta-opioid receptors are involved in the galanin-induced antinociception^17^. We also demonstrated that GalR1 is involved in the pain modulation in the brain^31^. The present study was performed to determine whether GalR1 is involved in galanin-induced antinociception in CeA in normal rats, and further to demonstrate the antinociceptive effects induced by intra-CeA injection of galanin in rats with neuropathy, as well as the galanin and GalR1 expression in CeA in normal rats and rats with neuropathy.

## Results

### Intra-CeA administration of galanin induced antinociceptive effects in normal rats

**Figure 1:**
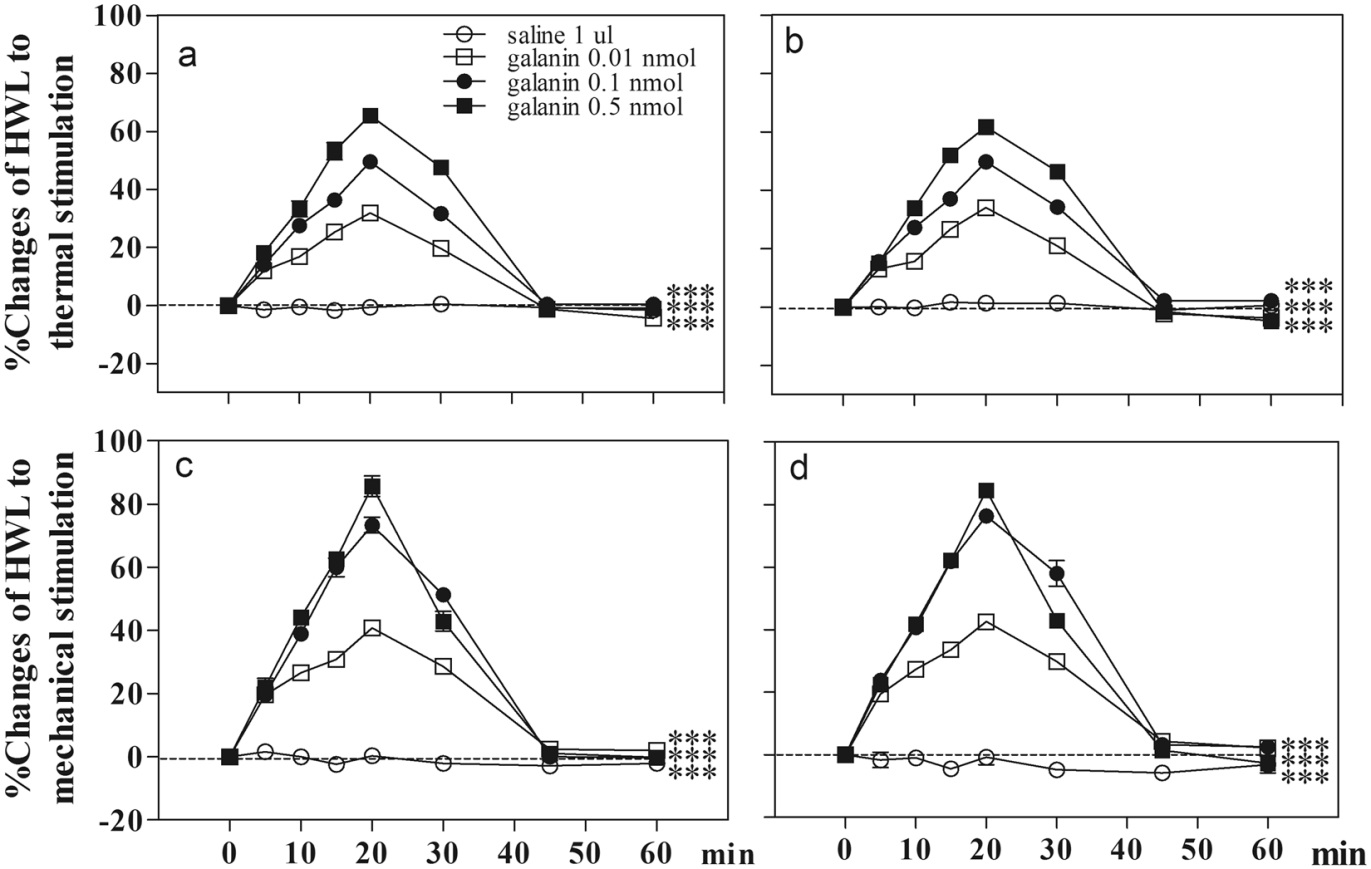
Effects of intra-CeA injection of galanin on the HWLs to thermal and mechanical stimulation in normal rats. Left HWL: (**a**) and (**c**); right HWL: (**b**) and (**d**). CeA, the central nucleus of amygdala; HWL, hindpaw withdrawal latency. Data are presented as mean ± S.E.M. Two-way ANOVA. ***P < 0.001.

Four groups of normal rats received intra-CeA injection of 0.01 (n = 8), 0.1 (n = 8) or 0.5 nmol (n = 8) of galanin, or 1 µl of 0.9% saline as a control (n = 8). The results are shown in Fig. 1.

As shown in Fig. 1, the hindpaw withdrawal latencies (HWLs) to noxious thermal and mechanical stimulations increased significantly in a dose-dependent manner after intra-CeA injection of 0.01 nmol of galanin (Hot-plate Test: F_left/left_ = 109.27, P < 0.001; F_right/right_ = 95.53, P < 0.001; Randall Sellitto Test: F_left/left_ = 234.48, P < 0.001; F_right/right_ = 253.12, P < 0.001), 0.1 nmol of galanin (Hot Plate Test: F_left/left_ = 159.65, P < 0.001; F_right/right_ = 159.39, P < 0.001; Randall Sellitto Test: F_left/left_ = 165.54, P < 0.001; F_right/right_ = 206.05, P < 0.001), 0.5 nmol of galanin (Hot Plate Test: F_left /left_ = 153.29, P < 0.001; F_right/right_ = 131.20, P < 0.001; Randall Sellitto Test: F_left/left_ = 160.31, P < 0.001; F_right/right_ = 172.09, P < 0.001) compared with the control group. The results demonstrated that intra-CeA injection of galanin induced significant antinociceptive effects in normal rats.

### Inhibitory effects of galanin receptor antagonist M40 on galanin-induced antinociception in normal rats

**Figure 2:**
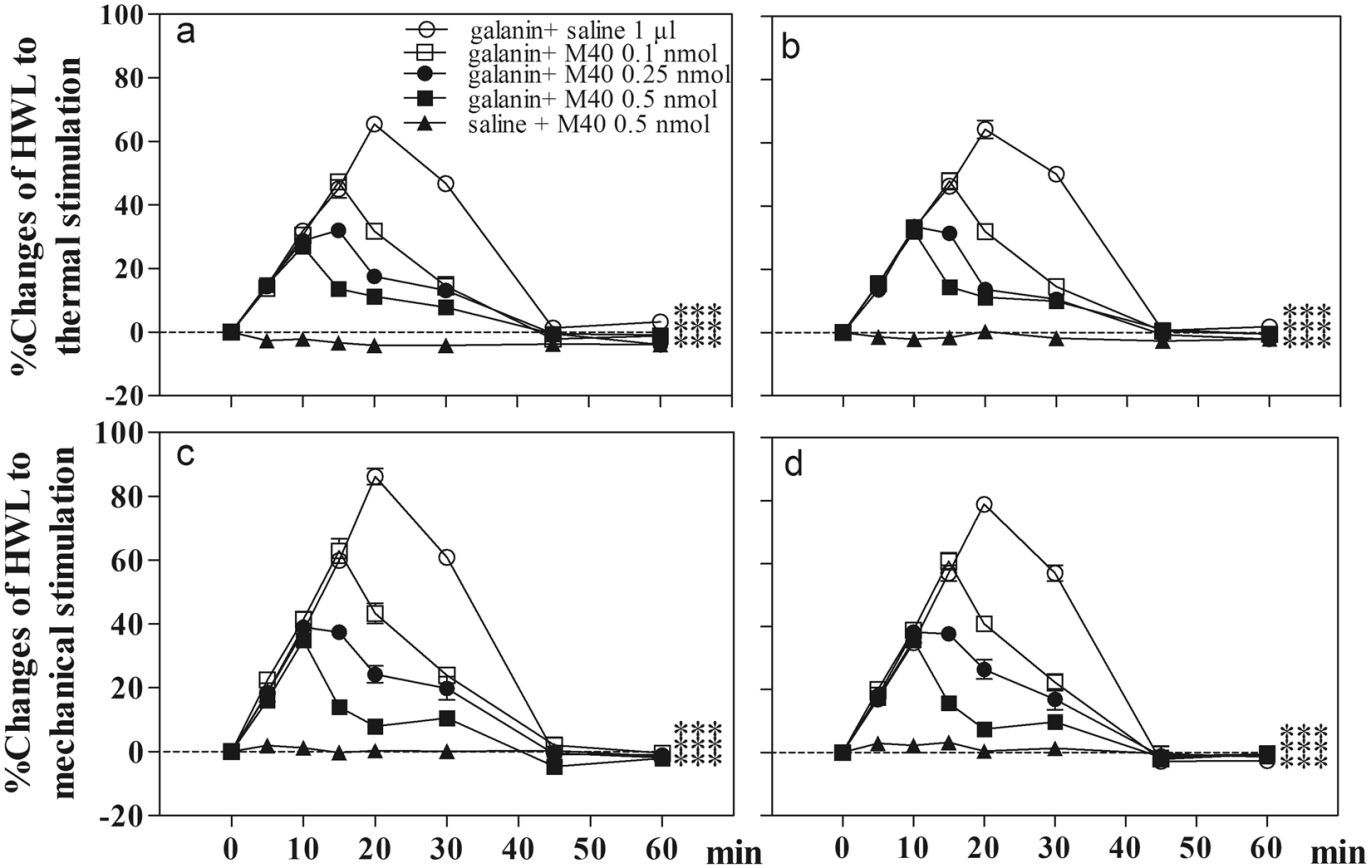
Effects of intra-CeA injection of M40 on the galanin-induced increases in HWLs to thermal and mechanical stimulation in normal rats. Left HWL: (**a**) and (**c**); right HWL: (**b**) and (**d**). Time = 0 min: intra-CeA injection of 1 nmol of galanin; time = 5 min: intra-CeA injection of 0.1 nmol, 0.25 nmol, 0.5 nmol of M40, or 1 μl of 0.9% saline as a control. Two-way ANOVA, **P < 0.01, ***P < 0.001.

To determine the involvement of galanin receptor in galanin-induced antinociception in CeA of normal rats, four groups of rats received intra-CeA injection of 0.5 nmol galanin, followed 5 min later by intra-CeA injection of 0.1 nmol (n = 7), 0.25 nmol (n = 7) or 0.5 nmol (n = 8) of galanin receptor antagonist M40, or 1 µl of 0.9% saline as a control (n = 8). After intra-CeA administration of galanin, the HWL increased. The galanin-induced increases in HWLs were significantly attenuated after intra-CeA injection of 0.1 nmol of M40 (Hot Plate Test: F_left/left_ = 39.27, P < 0.001; F_right/right_ = 30.57, P < 0.001 Randall Sellitto Test: F_left/left_ = 20.19, P < 0.001; F_right/right_ = 20.43, P < 0.001), 0.25 nmol of M40 (Hot Plate Test: F_left/left_ = 71.18, P < 0.001; F_right/right_ = 56.77, P < 0.001; Randall Sellitto Test: F_left/left_ = 50.69, P < 0.001; F_right/right_ = 39.58, P < 0.001), and 0.5 nmol of M40 (Hot Plate Test: F_left/left_ = 91.04, P < 0.001; F_right/right_ = 74.63, P < 0.001; Randall Sellitto Test: F_left/left_ = 84.42, P < 0.001; F_right/right_ = 63.75, P < 0.001), compared with the saline group. These results indicate that galanin receptors are involved in the galanin-induced antinociception in the CeA of normal rats, as shown in Fig. 2.

Another group of rats (n = 8) received intra-CeA injection of 1 µl of 0.9% saline, followed 5 min later by intra-CeA injection of 1nmol of M40. The results showed that there were no marked changes in HWLs to thermal and mechanical stimulation after intra-CeA administration of the galanin receptor antagonist M40, as shown in Fig. 2.

The results indicate that galanin receptors are involved in the galanin-induced antinociception in the CeA of normal rats.

### Activation of galanin receptor 1 by M617 in CeA induced antinociception in normal rats

**Figure 3:**
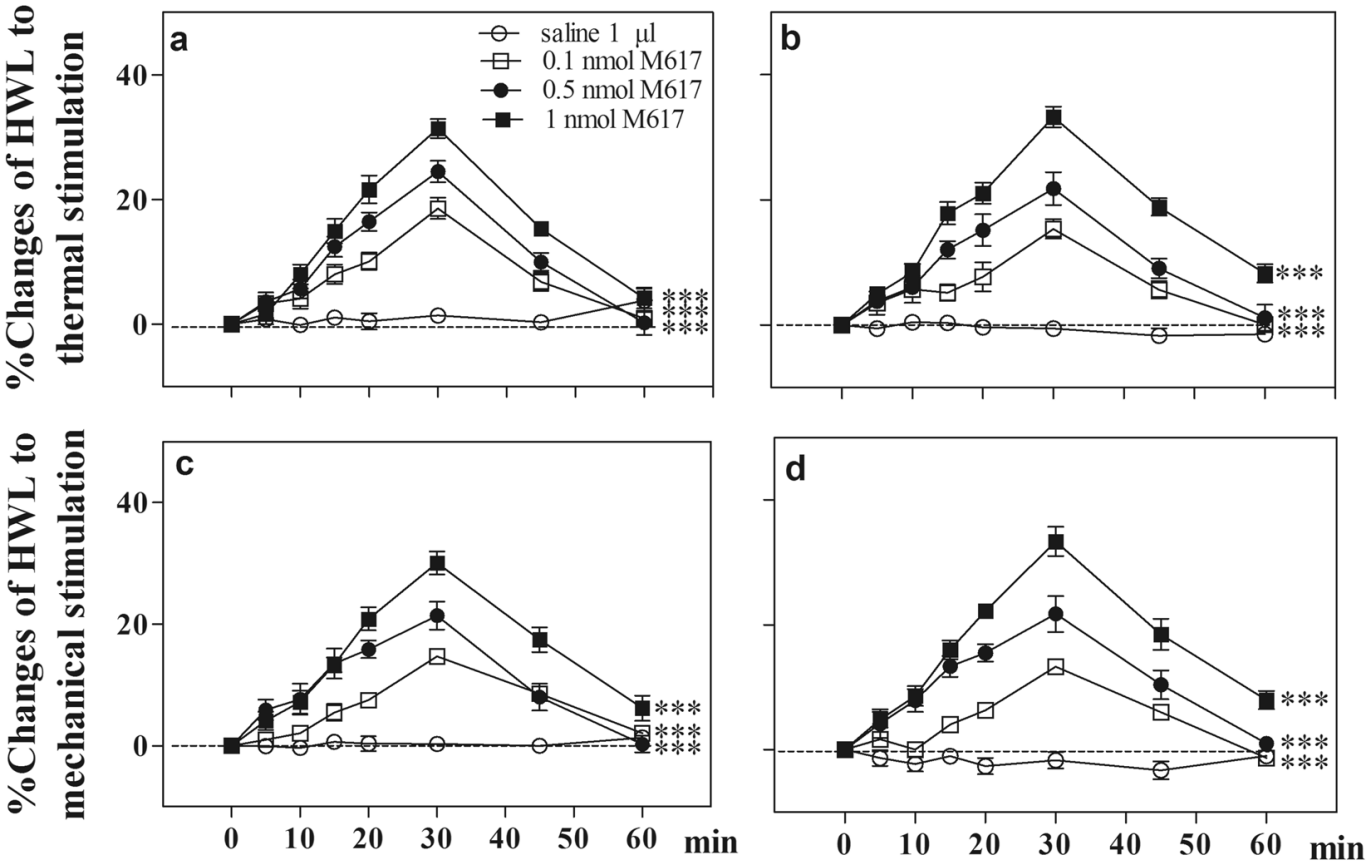
Effects of intra-CeA injection of the GalR1 agonist M617 on the HWLs to noxious thermal and mechanical stimulation in normal rats. Hot plate test: (**a**), left; (**b**), right. Randall Selitto test: (**c**), left; (**d**), right. Two-way ANOVA, ***P < 0.001.

To further determine the involvement of GalR1 in nociceptive modulation in CeA, the Gal R1 agonist M617 was used. Four groups of normal rats received intra-CeA injection of 0.1 (n = 6), 0.5 (n = 6) or 1 nmol (n = 6) of M617, or 1 µl of 0.9% saline as a control (n = 6). The results are shown in Fig. 3.

As shown in Fig. 3, the HWLs to noxious thermal and mechanical stimulations increased significantly in a dose-dependent manner after intra-CeA injection of 0.1 nmol of M617 (Hot-plate Test: F_left/left_ = 45.04, P < 0.001; F_right/right_ = 65.72, P < 0.001; Randall Sellitto Test: F_left/left_ = 61.98, P < 0.001; F_right/right = _63.44, P < 0.001), 0.5 nmol of M617 (Hot Plate Test: F_left/left_ = 63.13, P < 0.001; F_right/right_ = 101.02, P < 0.001; Randall Sellitto Test: F_left/left_ = 82.82, P < 0.001; F_right/right_ = 119.82, P < 0.001), or 1 nmol of M617 (Hot Plate Test: F_left/left_ = 88.48, P < 0.001; F_right/right_ = 189.27, P < 0.001; Randall Sellitto Test: F_left/left_ = 127.16, P < 0.001; F_right/right_ = 182.86, P < 0.001) compared with the control group. The results suggest that GalR 1 may be involved in the galanin-induced nociceptive modulation in CeA in normal rats.

### Involvement of PKC in the galanin-induced antinociception in CeA

**Figure 4:**
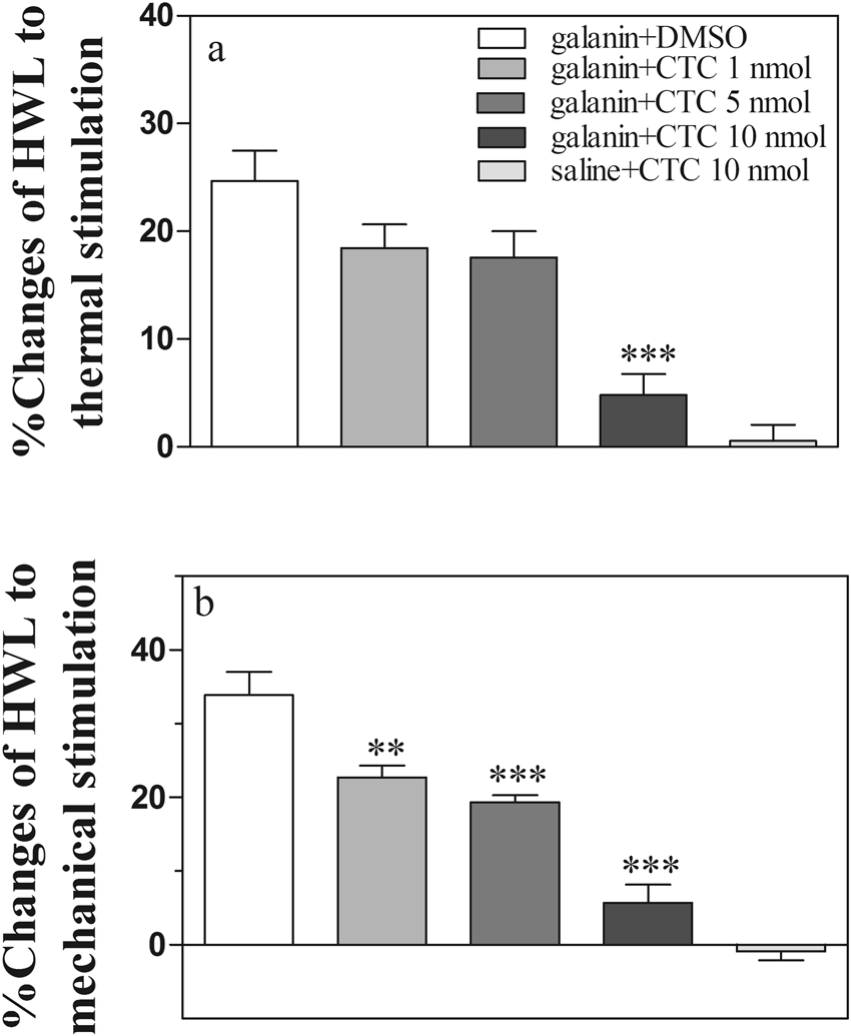
Effects of intra-CeA injection CTC on the galanin-induced antinociception in normal rats. (**a**) Hot plate test; (**b**) Randall Selitto test. CTC, chelery thrine chloride, a PKC inhibitor. One-way ANOVA followed Bonferroni test. **P < 0.01 and ***P < 0.001.

To study the involvement of PKC in the galanin-induced antinociception, the PKC inhibitor chelery thrine chloride (CTC) was used. Four groups of rats received intra-CeA injection of 0.5 nmol of galanin, followed 5 min later by intra-CeA injection of 1 nmol (n = 8), 5 nmol (n = 8), 10 nmol of CTC (n = 8), or 1 μl of DMSO (n = 8) as a control. The results are shown in Fig. 4.

After intra-CeA injection of galanin, the HWLs to noxious thermal and mechanical stimulation increased markedly. Then the HWLs decreased significantly after intra-CeA administration of 1 nmol of CTC (Hot-plate Test: P = 0.44; Randall Sellitto Test: P < 0.01), 5 nmol of CTC(Hot-plate Test: P = 0.26; Randall Sellitto Test: P < 0.001), or 10 nmol of CTC (Hot-plate Test: P < 0.001; Randall Sellitto Test: P < 0.001), compared with control group tested by one-way ANOVA followed Bonferroni test. The results demonstrated that inhibiting the PKC activity by CTC in CeA reduces the galanin-induced antinociception in normal rats, indicating that PKC may be involved in the galanin-induced antinociception in CeA in normal rats.

Another group of rats (n = 8) received intra-CeA injection of 1 μl of 0.9% saline, followed 5 min later by intra-CeA injections of 10 nmol of CTC (n = 8). There are no marked changes in HWLs to noxious thermal and mechanical stimulation after injection of CTC. The results are shown in Fig. 4.

### Antinociceptive effects induced by intra-CeA administration of galanin in rats with neuropathy

**Figure 5:**
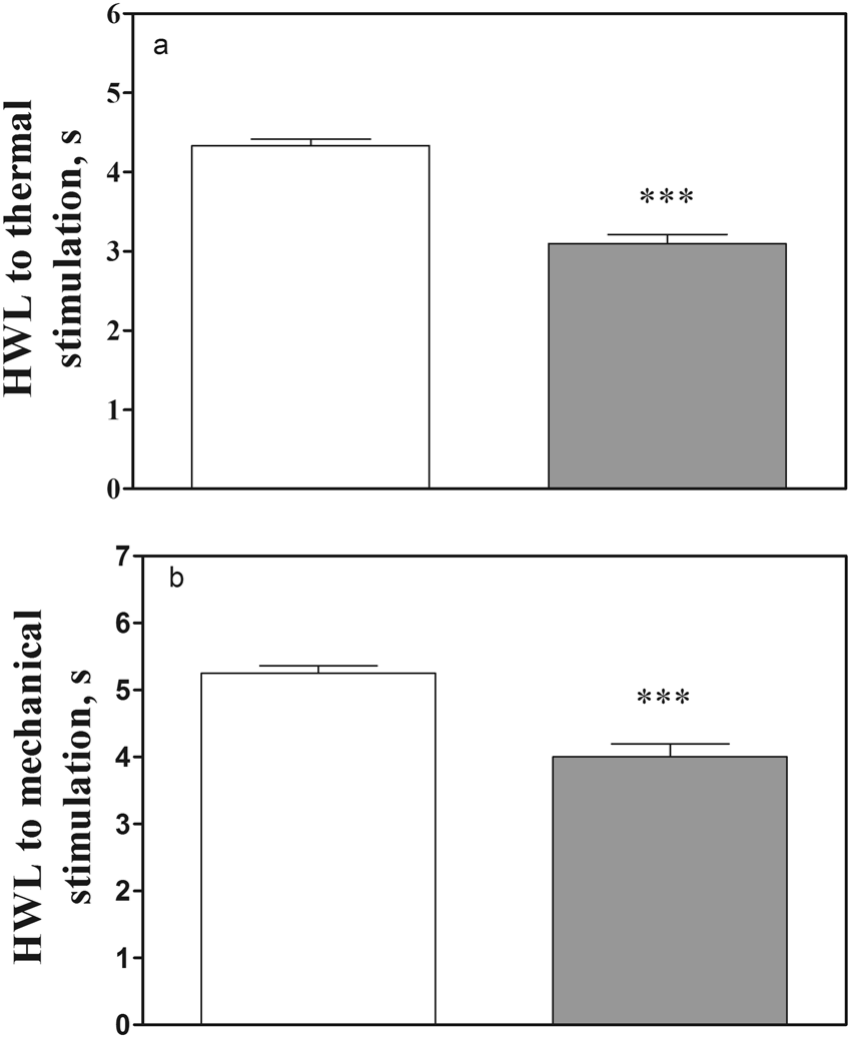
Comparison of the basal left HWLs in normal rats and rats with neuropathic pain. (**a**) Hot plate test; (**b**), Randall Selitto Test. Student’s t-test (two tails), ***P < 0.001.

In order to determine the influence of galanin on neuropathic pain, rats received left sciatic nerve ligation. The basal left HWL decreased significantly **(**Hot-plate Test: t = 8.74, P < 0.001**;** Randall Selitto Test: t = 5.57, P < 0.001**)** in rats with neuropathic pain 10 days after the surgery compared with normal rats, the results are as shown in Fig. 5.

**Figure 6:**
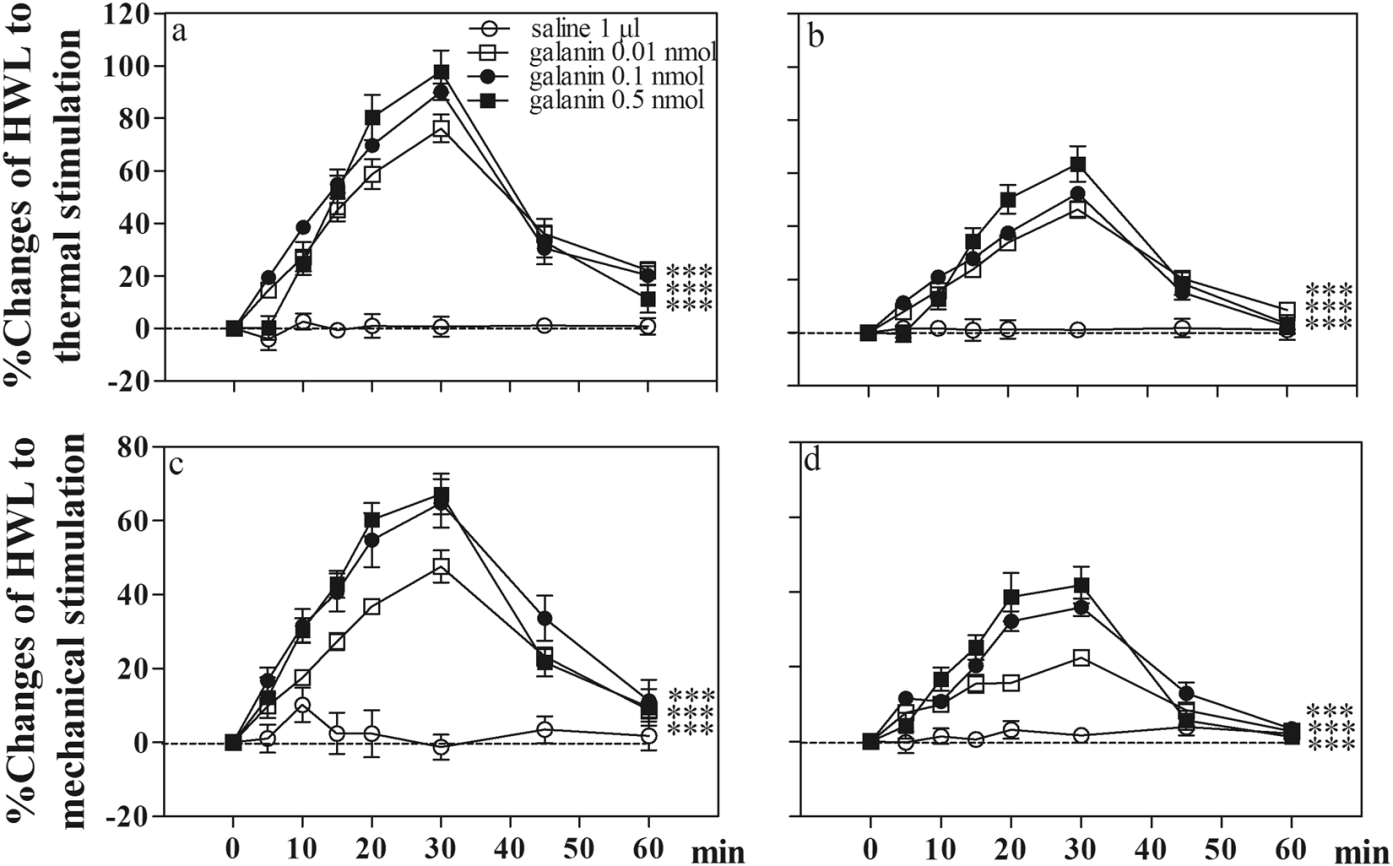
Intra-CeA injection of galanin induced antinociception in rats with neuropathic pain. (**a**) and (**b**), hot plate test; (**c**) and (**d**), Randall Selitto Test. (**a**) and (**c**): left HWL; (**b**) and (**d**): right HWL. Two-way ANOVA, ***P < 0.001.

Four groups of rats with sciatic nerve ligation received intra-CeA injection of 0.01 (n = 8), 0.1 (n = 7), or 0.5 nmol (n = 8) of galanin, or 1 μl of 0.9% saline as a control (n = 7). The results are shown in Fig. 6.

The HWLs to noxious thermal and mechanical stimulations increased significantly in rats with neuropathy after intra-CeA injection of 0.01 nmol of galanin (Hot-plate Test: F_left/left_ = 191.75, P < 0.001; F_right/right_ = 116.24, P < 0.001; Randall Selitto Test: F_left/left_ = 82.51, P < 0.001; F_right/right_ = 79.96, P < 0.001), 0.1 nmol of galanin (Hot-plate Test: F_left/left_ = 230.53, P < 0.001; F_right/right_ = 101.02, P < 0.001; Randall Selitto Test: F_left/left_ = 100.88, P < 0.001; F_right/right_ = 106.38, P < 0.001), or 0.5 nmol of galanin (Hot-plate Test: F_left/left_ = 90.24, P < 0.001; F_right/right_ = 61.45, P < 0.001; Randall Selitto Test: F_left/left_ = 96.30, P < 0.001; F_right/right_ = 56.26, P < 0.001) compared with the control group. The results demonstrated that intra-CeA injection of galanin induced significant antinociceptive effects in rats with neuropathy.

### Changes of the mRNA levels and the content of galanin in CeA in rats with neuropathic pain

**Figure 7:**
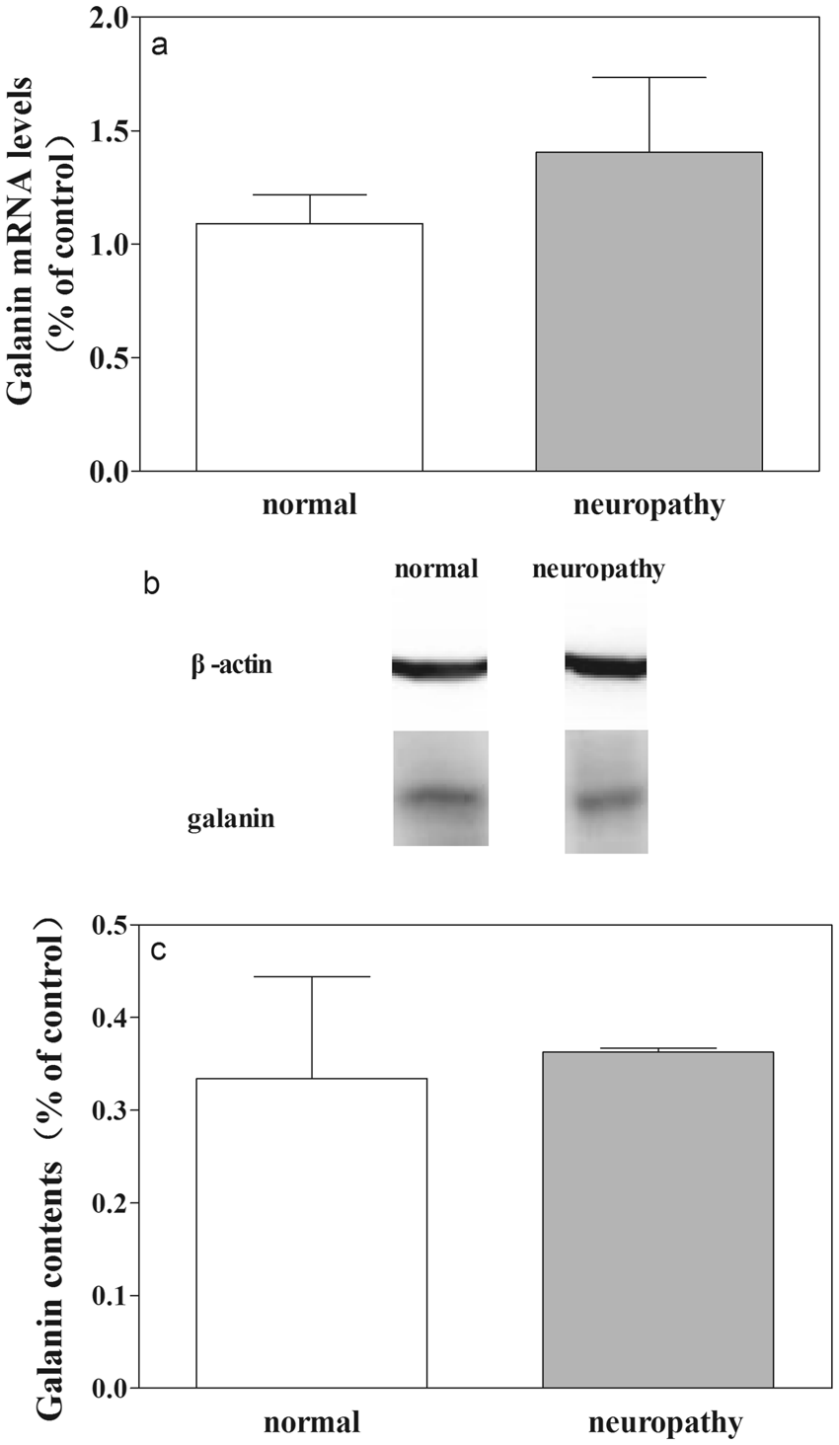
Influences of neuropathic pain on the expression of galanin mRNA levels and galanin content in CeA. (**a**) Results from RT-PCR test; (**b**) and (**c**), results from western blot test. Student’s t-test (two tails), *P < 0.05.

The galanin mRNA levels in CeA in normal rats (n = 3) and in rats with neuropathy (n = 3) were tested by real-time PCR (RT-PCR) and the results are shown in Fig. 7a. There are no significant changes in the galanin mRNA level (t = 0.89, P = 0.38) in CeA in rats with neuropathy than that in normal rats. The results indicate that there are no significant changes in galanin expression in CeA in rats with neuropathic pain.

We further determined the influence of neuropathic pain on the content of galanin in CeA. The contents of galanin in CeA in normal rats (n = 3) and rats with neuropathy (n = 3) were tested by western blot and the results are shown in Fig. 7b and c. There are no significant changes in the content of galanin in CeA (t = 0.26, P = 0.81) in rats with neuropathy than that in normal rats.

The above results indicate that there are no significant changes in mRNA levels and the content of galanin in CeA in rats with neuropathic pain compared to normal rats.

### Changes of the mRNA levels and the content of GalR1 in CeA in rats with neuropathic pain

**Figure 8:**
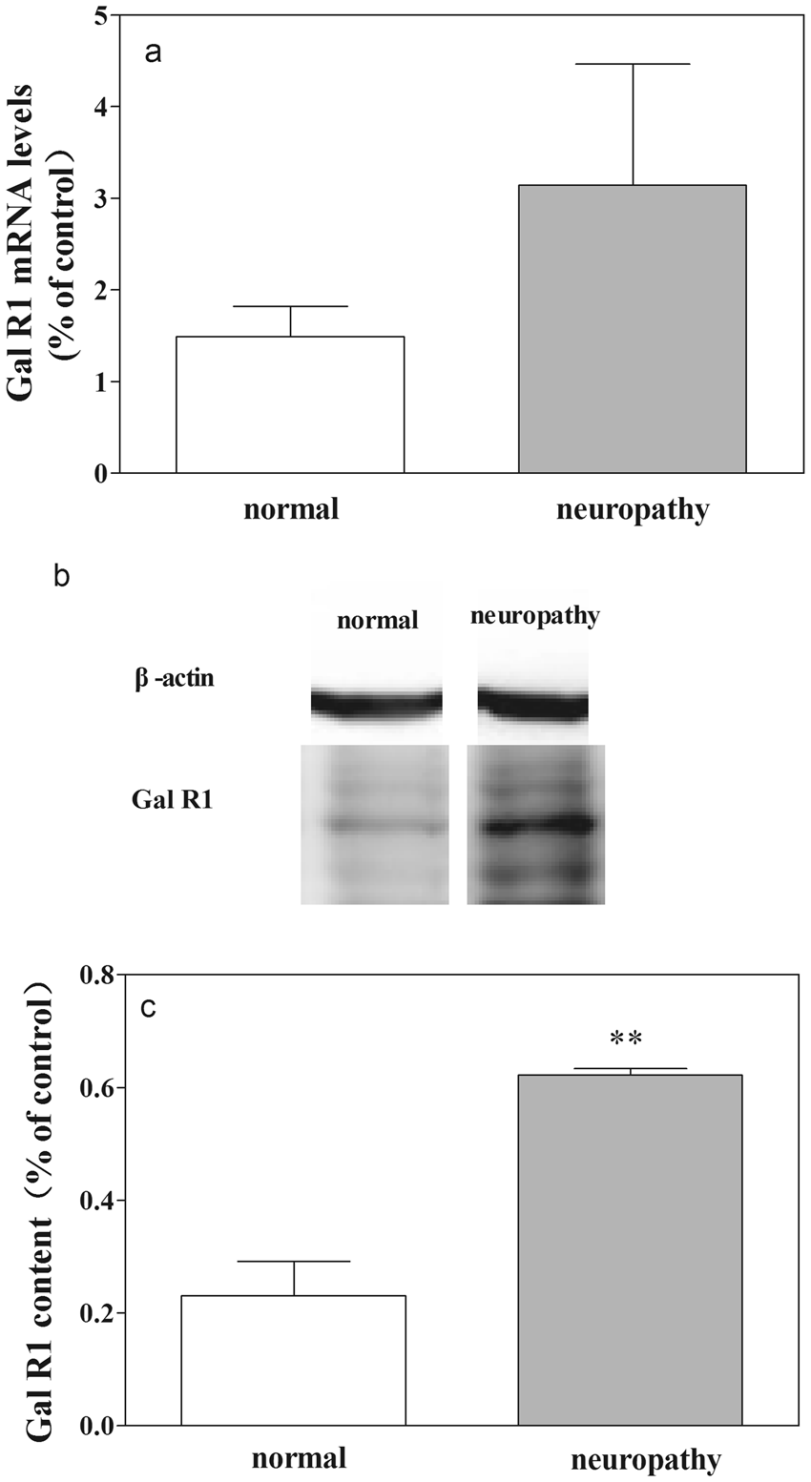
Influences of neuropathic pain on the expression of GalR1 mRNA levels and GalR1 content in CeA. (**a**) Results from RT-PCR test; (**b**) and (**c**), results from western blot test. Student’s t-test (two tails), **P < 0.01.

Our results have demonstrated that GalR1 is involved in the galanin-induced antinociception in the CeA in normal rats and rats with neuropathy. It is interesting to find the influences neuropathic pain on the expression of GalR1 in CeA of rats. The mRNA levels of GalR1 in CeA and the content of GalR1 in CeA in normal rats and rats with neuropathy were determined by RT-PCR and western blot, and the results are shown in Fig. 8.

As shown in Fig. 8a, there is an increased trend in the mRNA level of GalR1 (t = 1.04, P = 0.30) in CeA in rats with neuropathy than that in normal rats tested by RT-PCR. The results indicate an increased trend in the mRNA level of GalR1 in CeA in rats with neuropathic pain.

Figure 8b and c show the changes of GalR1 content in CeA in normal rats and in rats with neuropathy. There is a significant increase in the content of GalR1 (t = 6.24, P < 0.01) in CeA in rats with neuropathy than that in normal rats tested by western blot. The above results indicate that the expression of GalR1 increased in CeA in rats with neuropathic pain.

## Methods

### Animal preparations

All experiments were carried out on freely moving male Sprague–Dawley rats weighing 220 to 260 g (Experimental Animal Center of Luye Pharmaceutical Company, Yantai, China). The rats were housed in cages with free access to food and water, and maintained in a room temperature of 20 ± 2 °C with a 12 h/12 h light/dark cycle. All experiments were performed according to the guidelines of the International Association for the Study of Pain^32^ and the Guidelines for the Care and Use of Laboratory Animals of Yantai University. Our experiments were approved by Laboratory Animal Ethics Committee of Yantai University and the authorization number is 20140901-01. And every effort was made to minimize both the animal suffering and the number of animals used.

### Chemicals for intra-CeA injection

Solutions for intra-CeA injection were prepared with 0.9% sterilized saline, each with a volume of 1 µl containing: (1) 0.01, 0.1 or 0.5 nmol of galanin (rat galanin, 1–29 amino acids, Tocris, UK); (2) 0.1, 0.25, 0.5 or 1 nmol of M40 (M40, Tocris, UK); (3) 0.1, 0.5 or 1 nmol of M617 (M617, Tocris, UK); (4) 1, 5, or 10 nmol of chelery thrine chloride (chelery thrine chloride, the PKC inhibitor, Tocris, UK).

### Nociceptive tests

Rats were accustomed to the test condition for 3 days before the experiment to minimize the stress induced by handling and measurements. The hindpaw withdrawal latencies (HWLs) during thermal and mechanical stimulation were measured as described previously^17,18,23,24^. Briefly, the entire ventral surface of the rat hindpaw was placed manually on a hot plate (YLS-6B Intelligent Heat Panel Instrument, Jinan Yiyan Science & Technology Development Co., Ltd., Jinan, China), which was maintained at a temperature of 52 ± 0.2 °C. The time to hindpaw withdrawal was measured in seconds and referred to as the HWL to thermal stimulation. The Randall Selitto Test (Ugo Basile, Type 7200, Italy) was used to assess the HWL to mechanical stimulation. A wedge-shaped pusher at a loading rate of 30 g/s was applied to the dorsal surface of the hindpaw. The latency required to initiate the withdrawal response was assessed and expressed in seconds. Before intra-CeA injection, the HWLs were tested three times and regarded as the basal HWLs. The HWLs recorded during subsequent experiments were expressed as percentage changes of the basal level for each rat (% changes of the HWL). Each rat was tested by both types of stimulation. Every measurement of the HWL to both thermal and mechanical stimulation was finished within 1–2 min. A cut-off limit of 15 s was set up to avoid tissue damage.

### Surgical procedures and intra-CeA injection

Rats were anaesthetized by intraperitoneal injection of pentobarbital sodium (50 mg/kg, Xudong Chemical Factory, Beijing, China) and mounted on stereotaxic frame, a stainless steel guide cannula of 0.8 mm outer diameter was directed to the CeA (2.3 mm posterior to Bregma; 3.8 mm lateral to midline; 8 mm ventral to the surface of skull) according to Paxinos and Watson^33^, and was fixed to the skull by dental acrylic. There were more than 3 days for rats to recover from the operation. On the day of experiment, a stainless steel needle with 0.4 mm outer diameter was directly inserted into the guide cannula with 1.5 mm beyond the tip of the latter. One microliter of solution was thereafter infused into the CeA over 1 min. The injection needle was left in the site for 1 min after each injection before removal.

### The rat model of the neuropathic pain

The neuropathy model was produced as previously reported^16,18,23,24,34^. Animals were anesthetized by intraperitoneal injection of sodium pentobarbital (50 mg/kg) and 8–10 mm of the left common sciatic nerve was exposed at the level of the mid-high. Four loose ligatures were placed around the dissected nerve at 1.0–1.5 mm intervals. The ligations were carefully manipulated so that the nerve was barely constricted, and the skin incision was closed with silk sutures and animals allowed to recover.

### RT-PCR

The methods of RT-PCR were described in our published paper^24^. Briefly, normal rats (n = 3, as a control) and rats with sciatic nerve ligation 8 days after surgery (n = 3) received injection of over dose of 10% trichloroacetaldehyde monohydrate and the brain was removed immediately. The regions of CeA were dissected on ice and then frozen at −80 °C. The total RNA was isolated with TRIzon Reagent (CoWin Biosciences, Beijing,China). Total RNA was reverse-transcribed using SuperRT cDNA Synthesis Kit (CoWin Biosciences, Beijing, China). PCRs were done using UltraSYBR Mixture (CoWin Biosciences, Beijing, China). Sequences of primers for the experiments were rat galanin: sense 5′- CACATGCCATTGACAACCAC -3′ and antisense 5′-AACTCCATTATAGTGCGGACG-3′; rat galanin receptor 1: sense 5′-TCGGGACAGCAACCAAAC-3′ and antisense 5′-TGCAGATGATTGAGAACCTTGG-3′; rat GAPDH: sense 5′-GACCACCCAGCCCAGCAAGG-3′ and antisense 5′- TCCCCAGGCCCCTCCTGTTG -3′. The unigene expression levels were calculated with the 2−ΔΔCT method^35^.

### Western Blot

The methods of western blot were described in our published paper^24^. The normal rats (n = 3, as a control) and rats with sciatic nerve ligation 8 days after surgery (n = 3) received injection of over dose of 10% trichloroacetaldehyde monohydrate and the brain was removed immediately. The regions of the CeA were dissected on ice and then frozen at −80 °C. Total protein was extracted following our published protocol^24^, briefly the CeA samples homogenized in RIPA lysis buffer (Beyotime Institute of Biotechnology, Shanghai, China), the total protein was extracted and the whole protein extracts (80 µg) of CeA samples were subject to western blot assay. After transferred to PVDF membranes (Millipore, MA, USA), the membranes were incubated in blocking solution and then incubated with the polyclonal goat anti-galanin antibody (sc-16411, Santa Cruz Biotechnology, Inc, Santa Cruz, California, USA), polyclonal goat anti-GalR1 antibody (sc-16216, Santa Cruz Biotechnology, Inc, Santa Cruz, California, USA), beta-actin antibody (Beyotime Institute of Biotechnology, Shanghai, China) at 4 °C overnight. The membranes were washed with TBST and then probed with HRP-conjugated donkey anti-goat secondary antibody (Beyotime Institute of Biotechnology, Shanghai, China), HRP-conjugated goat anti-rabbit secondary antibody (Beyotime Institute of Biotechnology, Shanghai, China), HRP-conjugated goat anti-mouse secondary antibody (Beyotime Institute of Biotechnology, Shanghai, China). The brands were visualized by enhanced chemiluminescence (ECL) detection reagents (Beyotime Institute of Biotechnology, Shanghai, China) and imaged using ImageQuant LAS 4010 (GE Healthcare Bio-Sciences AB, Tokyo, Japan) and quantified using ImageQuant software (GE Healthcare Bio-Sciences AB, Tokyo, Japan).

### Statistical analysis

At the end of the experiments, the location of the tip of the injection needle was verified. Only the results obtained from nociceptive tests that the tips of the injection needle are within the CeA were used for statistical analysis. Data from the experiment were expressed as mean±S.E.M. Statistical difference between groups was determined by Two-way analysis of variance (ANOVA) for repeated measurements (F_left/left_ is the F value of the two groups: the left HWL of one group compared with the left HWL of another group); one-way ANOVA followed Bonferroni test, or Student’s t test (two-tailed). *P < 0.05, **P < 0.01 and ***P < 0.001 were considered as significant differences.

## Discussion

The CeA is a very important brain structure, and there are high densities of galanin and galanin receptors found in the CeA^3,4,6,7^. Lots of studies demonstrated that CeA is involved in pain modulation^17,28–30,36^. Gonçalves *et al*. found that systemic tapentadol resulted in dose-dependent decrease in right CeA neuronal activity only in neuropathy^28^. Plerre *et al*. reported that pain having a strong affective and emotional dimension, the amygdala, especially its CeA^36^. Recent research also demonstrated that CeA represents a convergence of pathway for pain, stress and emotion^30^. Previous studies have demonstrated that galanin and galanin receptors play important roles in the transmission and/or modulation of nociception in the central nervous system^6,11–24^. Wang and her colleagues demonstrated that administration of galanin into periaqueductal grey induced significant antinociceptive effects in intact rats and rats with neuropathy^20,21^. Jin *et al*. reported that galanin induced antinociceptive effects in the central nucleus of amygdala of rats, and opioid receptors are involved in the galanin-induced antinociception^17^. Sun and his colleagues reported that intra-arcuate nucleus of hypothalamus administration of galanin induced significant antinociceptive effects in rats^18^. In the present study, we found that intra-CeA injection of galanin induced dose-dependent increases in HWLs to thermal and mechanical stimulations in both normal rats and rats with neuropathy. These results suggest that the galanin plays an important role in nociceptive modulation in CeA in both normal rats and rats with neuropathy.

There are few studies demonstrated that galanin receptors are involved in the pain modulation in the brain. Fu and his colleagues found that intra-cerebroventricular inject the GalR1 agonist M 617 affect the antinociception in rats^15^. Kong and Yu have demonstrated that GalR1 is involved in the pain modulation in the brain^31^. Alier and his colleagues reported that GalR1 and GalR2 activated on both inhibitory and excitatory neurons in relation to the spinal antinociceptive and pro-nociceptive actions of galanin, and to the possible association of GalR1 with the inhibitory G-protein, Gi/o.^37^.

In the present study we found that intra-CeA injection of galanin receptor antagonist M40 significantly attenuated the galanin induced antinociception in CeA, indicating the involvements of galanin receptors in nociceptive modulation in CeA. As Fu and his colleagues found that intra-cerebroventricular injection of the GalR1 agonist M 617 affected the antinociception in rats^15^, we furthermore demonstrated that intra-CeA administration of the GalR 1 agonist M 617 induced increases in HWLs to thermal and mechanical stimulations in normal rats, suggesting that galanin may activate GalR 1 to induce nociception in CeA of rats.

Protein kinase C (PKC) is a common molecular in the cellular signal pathway of G-protein coupled receptor, and it has been demonstrated to be involved in pain modulation^38^. The present study further demonstrated that intra-CeA injection of the PKC inhibitor inhibited the galanin-induced antinociception, showing an involvement of PKC in the galanin-induced antinociception in CeA of rats.

Nerve injury-induced pain is a common disease in clinic^39^. A lot of studies have explored the mechanisms of nerve injury-induced pain using animal models with neuropathy^16,21,24,34^. Previous study in our laboratory found that administration of galanin to periaqueductal grey induced antinociception in rats with experimentally induced neuropathy^21^. Recently, Zhang and her colleagues reported that intra-anterior cingulate cortex injection of galanin induced significant increases in HWLs to noxious thermal and mechanical stimulations in rats with neuropathy, the increased HWLs were significantly attenuated by intra-anterior cingulate cortex injection of the GalR 2 antagonist M871, indicating that galanin induces antinociceptive effects in anterior cingulate cortex in rats with neuropathy, and GalR 2 is involved in galanin-induced antinociceptive effects in the anterior cingulate cortex^24^. In the present study we found that intra-CeA injection of galanin induced dose-dependent increases in HWLs to thermal and mechanical stimulations in rats with neuropathy. Interestingly, we found that GalR 1 agonist M 617 induced antinociceptive effects in normal rats, suggesting that galanin may activate GalR 1 to induce nociception in CeA of rats. Furthermore, we found that both galanin mRNA expression and galanin content showed no significant changes in CeA in rats with neuropathy than that in normal rats, while GalR 1 content increased significantly in CeA in rats with neuropathy than that in normal rats. These results strongly suggest that GalR 1 plays an important role in nociceptive modulation in CeA in rats with neuropathic pain.

The above results indicate that GalR 1 plays an important role in neuropathic pain. We further checked the influence of M40 on galanin-induced antinociceptive effects in CeA in rats with neuropathy. It was found that to inhibit the galanin-induced antinociception should use high dose of M40 (1 nmol) than that in normal rats (0.5 nmol) (unpublished data), supporting the results that GalR1 content in CeA significantly increased in rats with neuropathy than that of normal rats.

Taken together, the results of present study illustrate that galanin plays an important role in nociceptive modulation in CeA in rats with neuropathy, and there may be an up-expression of GalR 1 in CeA during neuropathic pain.
